# Phenotypic and Gene Expression Differences between DA, BN and WOKW Rats

**DOI:** 10.1371/journal.pone.0038981

**Published:** 2012-06-29

**Authors:** Jörn Lange, Thomas Barz, Axel Ekkernkamp, Barbara Wilke, Ingrid Klöting, Niels Follak

**Affiliations:** 1 Department of Trauma and Reconstructive Surgery, Medical Faculty, University of Greifswald, Greifswald, Germany; 2 Department of Orthopedic Surgery, Asklepios Clinic Uckermark, Schwedt, Germany; 3 Department of Laboratory Animal Science, Medical Faculty, University of Greifswald, Greifswald, Germany; 4 Orthopedic Clinic, Pfeiffersche Stiftungen, Magdeburg, Germany; Karolinska Institutet, Sweden

## Abstract

**Background:**

Because inbred rat strains are widely used as laboratory models, knowledge of phenotypic and genetic variations between strains will be useful to obtain insight into the relationship between different strains.

**Methods and Results:**

We studied phenotypic traits: of each strain – BN/K, DA/K and WOKW –10 male rats were studied for body weight and serum constituents at an age of 10 and 30 weeks. In addition, a total of 95 rats were studied for life expectancy. At an age of 30 weeks, these male rats were killed by an overdose of anesthetic (Sevofluran, Abbott), and the subcutaneous and visceral adipose tissue as well as bone tissue were removed to study the expression of 20 genes. There were significant differences in body weight, serum lipids and leptin at an age of 30 weeks between strains. Regarding life expectancy, BN rats lived longest (1072±228d). The highest gene expression was found in bone of BN rats. In adipose tissues, *Nfkb1* is only expressed in subcutaneous adipocytes, and 5 genes, *Col2a1, Mmp9, Tnfa, Ins1* and *Cyp24a1,* are not expressed in adipocytes. The ranking BN = DA>WOKW was observed in only one gene in subcutaneous (*Fto*) and visceral adipocytes (*Col6a1*). There were no significant differences in gene expression of one gene in subcutaneous adipocytes and of 3 genes in visceral adipocytes. Comparing the gene expression in visceral and subcutaneous adipocytes, only one gene showed a comparable behavior (*Bmp1*).

**Conclusion:**

From these results, it can be concluded that obvious phenotypic differences are caused by genetic differences between three rat strains, BN, DA and WOKW, as supported by gene expression studies in bone and adipose tissues. Especially BN rats can be used to study the genetic basis of long life.

## Introduction

Despite the recent advances in human genetics, animal models of human diseases remain attractive tools, not only to overcome genetic complexity, but also to permit studies under stable environmental conditions.

Rat inbred strains are widely used as laboratory models in understanding basic biology and human health and disease. Therefore, knowledge of phenotypic and genetic variation between strains will be useful to obtain insight into the relationship between different strains. This prompted us to study phenotypic traits and the expression of selected genes in three inbred rat strains to show the diversity of rat strains.

Brown Norway (BN) rats are slim, brown in color, have very soft fur, are susceptible to respiratory inflammation, but resistant to the induction of experimental allergic encephalomyelitis. They are also susceptible to the development of mercury-induced autoimmunity to renal basement membranes with the development of membraneous glomerulonephritis. It was BN rats that were used to encode the rat genome. Comparative studies showed that BN rats are obviously different in genome compared to several other inbred rat strains [1]–[3].

Dark agouti (DA) rats are agouti in colour, slim, and susceptible to allergic encephalomyelitis, collagen induced arthritis and other auto-immune diseases [4].

Wistar Ottawa Karlsburg RT1^u^ (WOKW) rats are white in color (albino), powerfully built and develop facets of metabolic syndrome with obesity, dyslipidemia, insulin resistance, hyperinsulinemia, hyperleptinemia and glucose intolerance [5]–[10]. Because of this phenotypic diversity, the aim of our study was to analyzed several phenotypic traits between BN, DA, and WOKW rats at an age of 10 and 30 weeks, and additionally we studied the life expectancy. Furthermore, these rat strains were also used for gene expression studies, as variation in gene expression is heritable and has been mapped to the genome in humans and model organisms [11]. Hence, we studied the expression of 20 selected genes in two organs, adipose tissue and bone; the genes selected play a role in bone and lipid metabolism as well as in immunologic reactions. These organs were chosen because DA and BN rats are slim and WOKW rats are obese and the bone architecture is different between DA and BN; BN rats tend to have fragile vertebrae, whereas DA rats primarily tend to have fragile femoral necks [12].

## Results

As shown in Table 1, most differences were observed between BN and WOKW as well as DA and WOKW at an age of 10 weeks. At an age of 30 weeks, these significant differences remained in body weight, serum triglycerides, leptin and insulin. The life expectancy was also significantly different: BN rats lived longest (1072±228d), followed by WOKW rats (810±158d), and DA rats (523±75d) had the shortest life expectancy.

**Table 1:** Phenotype of male BN, DA and WOKW rats at an age of 10 and 30 weeks (mean ± SD).

Traits	BN		DA		WOKW		P-values between strains atage 10 and 30 weeks					
Age (weeks)	10	30	10	30	10	30	BN vs.DA		BN vs. WOKW		DA vs.WOKW	
	n = 10	n = 10	n = 10	n = 10	n = 10	n = 10	10	30	10	30	10	30
Body weight (g)	203±7	303±12	216±6	299±44	320±15	532±51	<.05	n.s.	<.001	<.001	<.001	<.001
Subcutaneous fat (g)	2.2±0.46	4.6±0.60	2.8±0.13	3.0±0.78	3.8±0.41	4.9±0.32	<.001	<.001	<.001	n.s	<.001	<.001
Visceral fat (g)	1.8±0.16	4.3±0.61	1.5±0.16	2.6±0.48	4.1±0.46	4.2±0.86	n.s.	<.001	<.001	n.s	<.001	<.001
Fat ratio#	1.2±0.3	1.1±0.1	1.9±0.1	1.2±0.1	0.9±0.1	1.2±0.3	<.001	n.s.	<.001	n.s.	<.01	n.s.
Serum triglycerides (mmol/l)	1.3±0.3	1.5±0.1	0.71±0.2	0.78±0.2	3.5±0.4	6.1±0.5	<.001	<.001	<.001	<.001	<.001	<.001
Serum Cholesterol (mmol/l)	1.9±0.15	1.5±0.12	2.6±0.19	2.8±0.22	2.2±0.26	2.8±0.4	<.001	<.001	<.01	<.001	<.001	n.s.
HDL-C (mmol/l)	1.3±0.07	1.1±0.2	0.8±0.01	0.9±0.02	0.7±0.03	0.8±0.05	<.001	n.s.	<.001	n.s.	<.001	n.s.
Leptin (ng/ml)	2.8±0.9	2.2±0.8	2.8±0.7	4.3±1.1	5.8±2.1	9.4±2.2	n.s.	<.05	<.001	<.001	<.001	<.001
Insulin (ng/ml)	1.8±1.3	1.4±0.2	1.7±0.8*	1.8±0.8	3.2±0.9	5.6±2.0	n.s.	n.s.	<.05	<.001	<.01	<.001
Survival (days)	1072±228 (n = 30)		523±75 (n = 25)		810±158 (n = 40)		<.001		<.001		<.001	

#Amount of subcutaneous (s) divided by amount of visceral adipose tissue (v).

**Figure 1:**
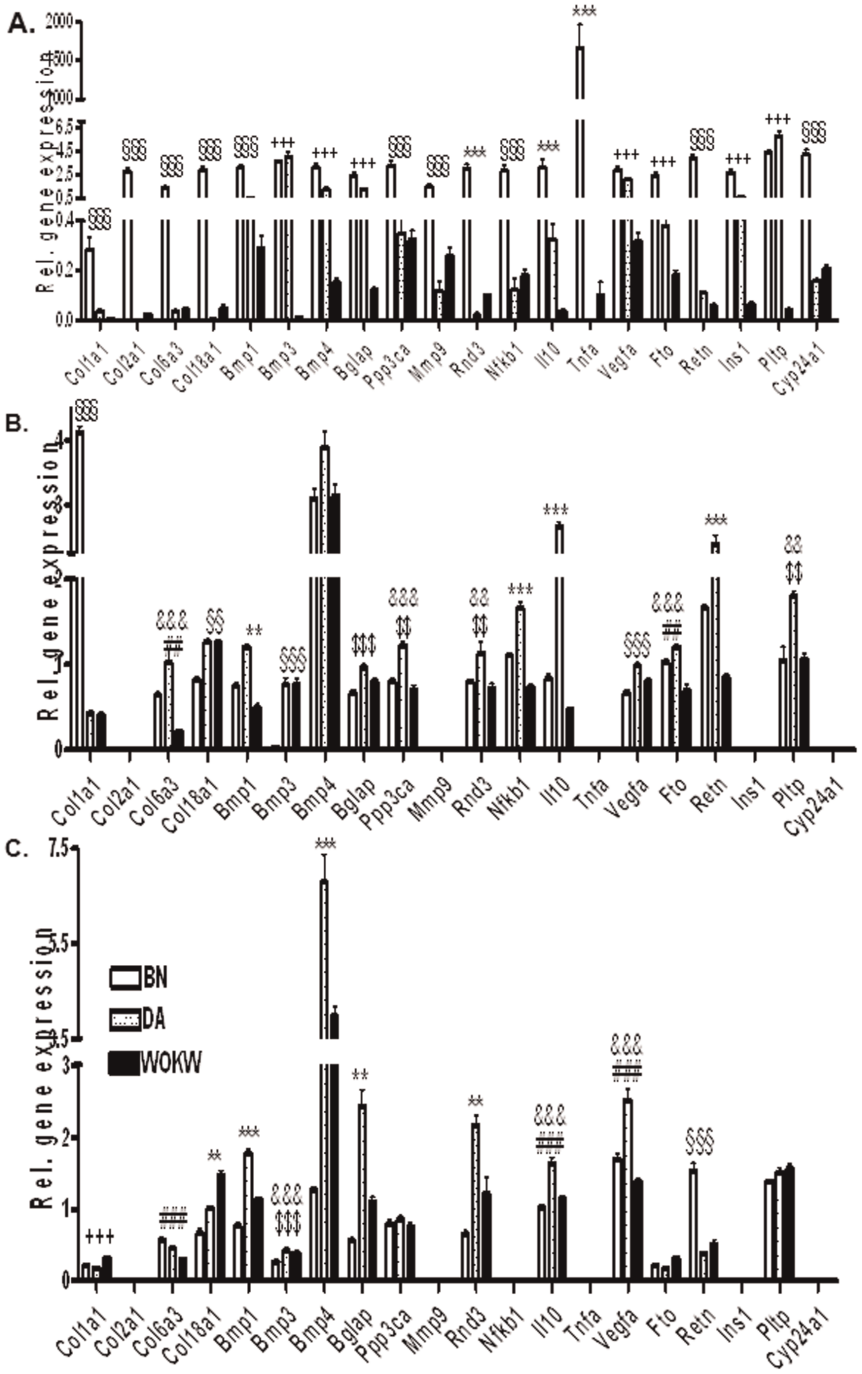
Relative gene expression in bone (A), subcutaneous (B) and visceral (C) adipocytes of 30 weeks old males (mean ± SEM). * BN, DA and WOKW are significantly different at 1 ** or 0.1*** per cent level. + BN and DA are significantly different to WOKW at 1++ or 0.1+++per cent level. § BN is significantly different to DA and WOKW at 1++ or 0.1+++per cent level. # BN is significantly different to WOKW at 1 ## or 0.1 ### per cent level. $ BN is significantly different to DA at 1 $$ or 0.1 $$$ per cent level. & DA is significantly different to WOKW at 1 && or 0.1 &&& per cent level.

The gene expression results in bone (A), subcutaneous (B) and visceral adipocytes (C) are demonstrated in Figure 1. In bone, BN rats showed the highest expression in 13 (*Col1a1, Col2a1,Col6a3,Col18a1, Bmp1, Ppp3ca, Mmp9, Rnd3, Nfkb, Il10, Tnfa, Retn, Cyp24a1*) out of 20 genes studied at an age of 30 weeks. In 7 genes (*Bmp3, Bmp4, Bglap, Vegfa, Fto, Ins1, Pltp*), the expression of BN rats was comparable to that of DA rats. It is obvious that the relative gene expression is higher in BN rats by factors of 10 (*Col6a3, Bglap, Nfkb1,Fto, Retn*), 20 (*Col2a1, Bmp4, Rnd3*), 30 (Col18a1) or even 140 (*Cyp24a1*) and 400 (*Tnfa*). Similar to BN, at an age of 30 weeks, a higher expression of *Bmp3, Vegfa* and *Pltp* is observed in DA rats by factors of 100, 7 and 17, respectively.

Analyzing the gene expression in adipose tissues, *Nfkb1* is only expressed in subcutaneous adipocytes, whereas 5 genes, *Col2a1, Mmp9, Tnfa, Ins1* and *Cyp24a1,* are not expressed in adipocytes but in bone. In subcutaneous adipocytes, 14 out of 15 genes are significantly different, whereas in visceral adipocytes 11 out of 15 genes showed significant differences. In contrast to bone, most genes in DA rats are more highly expressed than BN genes in subcutaneous adipocytes (13/15) and half of them are more highly expressed in visceral adipocytes (7/14). The ranking BN = DA>WOKW was observed in only one gene each in subcutaneous (*Fto*) and visceral adipocytes (*Col6a1*). Comparable behavior of gene expression was seen in *Col1a1* in bone and subcutaneous adipocytes (BN>DA = WOKW) and in *Bmp1* in both adipose tissues (BN<DA>WOKW). Furthermore, there were no significant differences in the expression of one gene (*Bmp4*) in subcutaneous adipocytes and of 3 genes (*Ppp3ca, Fto and Pltp*) in visceral adipocytes. There were also 2 genes (*Col1a1, Col18a1)* which showed a significantly higher expression in WOKW than in BN and DA rats in visceral adipocytes, which was never seen in subcutaneous adipocytes and bone. If the gene expression in visceral adipocytes is compared with those in subcutaneous adipocytes, only one gene showed a comparable expression (*Bmp1*).

In Table 2 correlations between fat ratio and gene expression in subcutaneous and visceral adipocytes are summarized. The fat ratio in BN rats correlated with gene expression of *Bmp3* (r = 0.892) and in DA rats with *Fto* expression (r = 0.924) in subcutaneous adipocytes. In WOKW rats, the fat ratio was negatively correlated with *Il10* (r = −0.896) and *Pltp* (r = −0.927) in subcutaneous adipocytes and positively in *Ppp3ca* (0.914) and *Pltp* (r = 0.864) in visceral adipocytes.

**Table 2:** Significant correlations between fat ratio and gene expression in subcutaneous (Sub) and visceral (Vis) adipocytes of BN, DA and WOKW rats.

Strains	Genes		Sub	Vis
DA	*Bmp3*	bone morphogenic protein3	0.892P = 0.017	
BN	*Fto*	fat mass and obesity associated	0.924P = 0.025	
WOKW	*Il10*	interleukin10	−0.896P = 0.016	
	*Pltp*	phospholipid transfer protein	−0.927P = 0.008	0.864P = 0.026
	*Ppp3ca*	protein phosphatase 3, catalytic subunit, alpha isoform		0.914P = 0.011

## Discussion

The aim the study was not only to show phenotypic differences between different rat strains but also to look at the DNA level by gene expression studies. The Phenotype of BN, DA and WOKW rats showed clearly the diversity of strains. The greatest differences were observed in serum triglycerides, which were significantly different between BN, DA and WOKW rats at an age of 10 and 30 weeks, and in life expectancy. The lowest triglyceride values were found in DA rats, followed by that of BN and finally WOKW rats.

However, the fat ratio also deserves our interest. Comparing the amount of subcutaneous and visceral fat it is obvious that the amount is increasing with age which is pronounced in BN rats where the amount increased more than 2 times in both, subcutaneous and visceral fat. The lowest increase or better a stagnation of visceral fat was observed in WOKW rats with age (4.1 vs. 4.2). Also taking serum leptin produced by adipocytes into consideration, the fat ratio remained similar in BN rats, but the leptin levels dropped. In DA rats, the fat ratio dropped in favor of visceral adipose tissue and serum leptin values increased. The reverse was found in WOKW rats. The fat ratio increased in favor of subcutaneous adipose tissue, and instead of decreasing, the leptin values increased obviously. That means the behavior between fat ratio and serum leptin values differs in BN, DA and WOKW rats.

Obvious differences were also found in serum cholesterol, in which the changes with age were interesting. From 10 to 30 weeks, the values decreased in BN (1.9–1.5 mmol/l), remained similar in DA (2.6–2.8 mmol/l) and increased in WOKW rats (2.2–2.8 mmol/l). This behavior between strains is comparable with those of serum insulin. Considering that a decrease in the total cholesterol as well as the low-density lipoprotein cholesterol level results in a reduction of the progression of vascular disease and of coronary heart disease, the cholesterol reduction in BN rats should result in a longer life which was found in BN rats [13], [14].

In terms of life expectancy, BN rats survived more than 1000 days, followed by WOKW and then DA rats. The latter had the lowest life expectancy, which was the half of BN rats. Given that BN rats have such a high life expectancy, their health should be excellent.

The behavior of some of these phenotypic traits should be reflected in gene expression in subcutaneous and visceral adipocytes.

The gene expression studies in adipose tissue of mice suggest that genes associated with vascularization and tissue remodelling are major regulatory points controlling differences in adipose tissue expansion. *Bmp3* and *Fto* belong to such genes showing a strong association with increased adiposity [15]. That may explain the high correlation between fat ratio and expression of *Bmp3* in DA and of *Fto* in BN rats. However, there were two different genes in BN and DA rats, suggesting differences in the regulation of body fat content.

In contrast to BN and DA rats, WOKW rats develop facets of metabolic syndrome [5]–[10]. This can explain the fact that more genes correlated with fat ratio in WOKW than in BN and DA. *Pltp* correlates with fat ratio which is positively correlated in visceral adipocytes and negatively in subcutaneous adipocytes. Considering the total values of fat the positively correlation in subcutaneous tissue is associated with an increase of fat from 10 to 30 weeks of age (3.8 vs. 4.9g) whereas in visceral fat the fat mass remained comparable between 10 and 30 weeks (4.1 vs. 4.2g). As shown in human studies *PLTP* mRNA levels in subcutaneous, but not in the visceral, adipose tissue were positively related to the BMI of the subjects. These results strongly suggest that visceral and subcutaneous adipocytes may have different properties in the production of bioactive molecules [16]. The protein encoded by this gene is one of at least two lipid transfer proteins found in plasma. The encoded protein transfers phospholipids from triglyceride-rich lipoproteins to high density lipoprotein (HDL). This protein is thought to be involved in cholesterol metabolism. Furthermore, it has been suggested that *PLTP* might play a role in the regulation of body fat content. The mRNA levels and the activity of *PLTP* have been consistently associated with obesity [17]–[21]. Therefore, it can be assumed that *Pltp* also modulates the level of adiposity in WOKW rats. Additional studies in humans and mice have shown that there is an association between *PLTP* genetic variants and obesity-related phenotypes [22]–[25]. Therefore, *Pltp* could be a candidate gene for obesity in WOKW rats.

Besides fat ratio, marked differences were especially observed in life expectancy between BN, DA and WOKW rats. The longevity gene candidates have generally fallen into inflammatory and immune-related factors. The factors involved in the inflammatory response deserve particular attention, since aging is known to be accompanied by several changes in the immune system. The adaptive immune branch, such as the various T-lymphocyte subpopulations, shows considerable decline (“immunosenescence”), while in contrast, the innate part of immunity displays a two- to four-fold increase in the production of proinflammatory cytokines and acute phase proteins (“inflammaging”) [26]–[30]. Therefore, successful aging devoid of co-morbidities depends on a combination of well-balanced anti- and proinflammatory responses or processes that are largely genetically modified. If a gene’s expression is associated with survival, the relationship should be positively (BN>WOKW>DA) or negatively (BN<WOKW<DA) correlated. Analyzing gene expression in bone and adipose tissue, only two genes can be found in bone which support this idea: *Tnfa* and *Rnd3*.

TNFa is a pleiotropic proinflammatory cytokine expressed primarily in stimulated monocytes/macrophages in response to microbes, mitogens and other cytokines. Its functions are related to antitumor activity, immune modulation and inflammatory host response, and it is also implicated in age-associated chronic diseases [31]. Based on these facts, the high expression in bone of BN rats may indicate that proinflammatory cytokines are also highly expressed in other organs and thus cause the long life expectancy of BN rats. That is not more a speculation because we analysed the expression of *Tnfa* in blood and liver in addition to bone. In blood the relative *Tnfa* expression was increased by factor 100 in BN (6876±1145) in comparison to DA (50±12) and WOKW (74±24). The differences in the liver were present too but no so pronounced as in blood. The relative expression of *Tnfa* in liver of BN rats (90±11) was significantly higher compared with DA (22±3) and WOKW rats (15±5). Now one could assume that high *Tnfa* expression is related to life expectation. This assumption is supported by findings of Napolioni et al. 2011. They have shown that a combination of functional SNPs within TNFa and another gene can influence life expectancy in a gender-specific manner. Especially males lived longer than females [31].

*Rnd3* is also of interest in this context. Rnd proteins are a subfamily of Rho GTPases involved in the control of actin cytoskeleton dynamics and other cell functions, such as motility, proliferation and survival. Furthermore, the high expression of *Rnd3* in bone of BN rats may suggest a general phenomenon. *Rnd3* KO mice have shown that it is essential for postnatal development, since *Rnd3* null mice die shortly after birth and show important structural defects, such as the absence of the common peroneal nerve, and notable motor and behavioral deficits. Therefore, this gene may support longer life expectancy [32].

In conclusion, obvious phenotypic differences are caused by genetic differences between the three rat strains, BN, DA and WOKW, as supported by gene expression studies in bone and adipose tissues. Especially BN rats can be used to study the genetic basis of long life.

## Materials and Methods

### Animals

BN/K, DA/K and WOKW rats were bred and kept in our own animal facility. All rats were specified pathogen free and had free access to food (Ssniff V132, R-Z, Soest, Germany: raw protein 21.2%; raw fat 3.8%; crude fiber 4.4%; metabolizable energy 13.3 MJ/kg) and acidulated water. Rats were kept in Macrolon type III cages (Ehret, Emmendingen, Germany) in groups of two and maintained on a 12∶12-h light/dark cycle (5 AM/5 PM).

### Phenotype Characterization

Ten male BN, DA and WOKW rats were studied for body weight and serum parameters (triglycerides, total cholesterol, HDL cholesterol, insulin, leptin) at an age of 10 and 30 weeks. At an age of 30 weeks, these male rats were killed by an overdose of anesthetic (Sevofluran, Abbott), and the subcutaneous and visceral adipose tissue and bone tissue were removed. Adipose tissue was weighed and the relative fat ratio was determined by dividing the weight of subcutaneous fat by that of visceral fat.

Blood samples were obtained between 7 and 9 am. Serum leptin and serum insulin were determined using a radioimmunoassay kit (Rat Leptin RIA Kit; Linco Research, St Charles, MO, USA) or ELISA (Rat Insulin ELISA; Mercodia, Uppsala, Sweden). Total serum cholesterol, HDL cholesterol and serum triglycerides were analyzed by lipid electrophoresis.

In addition, other BN (n = 30), DA (n = 25) and WOKW rats (n = 40) were observed up to their natural death to determine the life expectancy of each strain. However, if the rats appeared moribund before natural death, they were killed with an overdose of anesthetic (Sevofluran, Abbott).

### Ethics Statement

The animal research protocol was in accordance with the current version of the German Law on the Protection of Animals. The experimentation was approved by the Animal Ethical Committee of Greifswald and by the appropriate local authority (Landesveterinär- und Lebensmitteluntersuchungsamt, Rostock, Germany).

### RNA Isolation and cDNA Synthesis

Bone and adipose tissue from 6 of 10 males at an age of 30 weeks were used for gene expression studies. At the time of euthanasia, the tibial bone was harvested from the proximal metaphysis to the tibiofibular junction, excluding all cartilaginous and soft tissue. The tibias were snap frozen in liquid nitrogen and pulverized. Total RNA was extracted with Trizol (Qiagen, Hilden, Germany). After death, subcutaneous and visceral adipose tissue was also removed. Adipocytes were isolated by collagenase (1 mg/ml) digestion for 30 min. Total RNA of subcutaneous and visceral adipocytes was isolated with RNeasy Mini Kit (Qiagen, Hilden, Germany). Residual DNA was removed by DNase treatment (RNase-Free DNase Set; Qiagen, Hilden, Germany) according to the manufacturer’s instructions. A defined amount of purified RNA (1.5 µg) from organ samples was transcribed in cDNA and stored at −20°C until use, as detailed previously [9], [33].

### Real-time Quantitative PCR

Real-time PCR was performed using the ABI PRISM sequence detection system 7000 (Perkin–Elmer Applied Biosystems, Foster City, CA, USA) according to the manufacturer’s instructions, using ABI PRISM 7000 SDS Software Version 1.1. Reactions were performed with 12.5 µl SYBR-Green Master Mix (ABI), 0.4–2.0 µl of each primer (50 ng/µl), 6 µl template (cDNA) or no template (NTC), and RNase-free water was added to obtain a final volume of 25 µl. The cycling conditions were as follows: 50°C for 2 min, initial denaturation at 95°C for 10 min, followed by 45 cycles of 95°C for 15 s, and 60°C for 1 min. Each quantitative PCR was performed in duplicate. Target cDNA was amplified by primer sets for collagen, type I, alpha 1 (*Col1a1*, Acc.-No. NM_053304.1), collagen, type II, alpha 1 (*Col2a1*, Acc.-No. NM_012929.1), procollagen, type VI, alpha 3 (*Col6a3*, Acc.-No. NM_001109008.1), collagen, type XVIII, alpha 1 (*Col18a1*, Acc.-No. XM_241632.5), bone morphogenetic protein 1 (*Bmp1*; Acc.-No. NM_031323.1), bone morphogenetic protein 3 (*Bmp3*, Acc.-No. NM_017105.1), bone morphogenetic protein 4 (*Bmp4*, Acc.-No. NM_012827.2), bone gamma-carboxyglutamate (gla) protein (*Bglap*, Acc.-No. NM_013414.1), protein phosphatase 3, catalytic subunit, alpha isoform (*Ppp3ca*, Acc.-No. NM_017041.1), matrix metallopeptidase 9 (*Mmp9*, Acc.-No. NM_031055.1), Rho family GTPase 3 (*Rnd3*, Acc.-No. NM_001007641.1), nuclear factor of kappa light polypeptide gene enhancer in B-cells 1(*Nfkb1*, Acc.-No. XM_001075876.2), interleukin 10 (*Il10*, Acc.-No. NM_012854.2), tumor necrosis factor alpha (*Tnfa*, Acc.-No. NM_012675.3), vascular endothelial growth factor A (*Vegfa*, Acc.-No. NM_001110336), fat mass and obesity associated gene (*Fto*, Acc.-No. NM_001039713.1), Resistin (*Retn*, Acc.-No. NM_144741.1), Insulin1 (*Ins1*, Acc.-No. NM_019129.3), phospholipid transfer protein (*Pltp*, Acc.-No. NM_001168543.1), and cytochrome P450, family 24, subfamily a, polypeptide 1 (*Cyp24a1*, Acc.-No. NM_201635.2). The rat 18S rRNA gene served as the endogenous reference gene. For each experimental sample, the amounts of targets and endogenous reference (18S rRNA) were determined from the calibration curve. The target amount was then divided by the endogenous reference amount to obtain a normalized target value.

### Statistical Analysis

Results are expressed as means ± SD (Table 1) or means ± SEM (Figure 1). Differences were assessed by one-way analysis of variance corrected by Bonferroni-Holm using the Statistical Package for the Social Sciences (SPSS, Chicago, IL, USA).
